# Supplementary material for: On the effect of phylogenetic correlations in coevolution-based contact prediction in proteins
Source: PLoS Comput Biol. 2021 May 24;17(5):e1008957. doi: 10.1371/journal.pcbi.1008957 (PMC8177639; doi:10.1371/journal.pcbi.1008957)
Supplement: S1 Text — (PDF) [file pcbi.1008957.s001.pdf]

Supporting information S1 Text: On the effect of phylogenetic correlations in coevolution-based contact prediction in proteins

Edwin Rodriguez Horta[1,2], Martin Weigt [2,*],

1 University of Havana, Physics Faculty, Department of Theoretical Physics, Group of Complex Systems and Statistical Physics, Havana, Cuba
2 Sorbonne Université, CNRS, Institut de Biologie Paris-Seine, Laboratoire de Biologie Computationnelle et Quantitative – LCQB, Paris, France

* martin.weigt@sorbonne-universite.fr

The supporting information contains the supplementary tables and figures.

Pfam ID	L	M	PDB ID
PF02906	243	4549	1feh
PF11976	72	2467	5gjl
PF00786	59	2747	1f3m
PF00988	128	8611	5dot
PF00338	98	6983	2mew
PF00617	177	7173	3t6a
PF02787	122	9080	1a9x
PF02609	52	6068	1vp7
PF10369	74	6405	2f1f

Table A. Protein families and PDB structures used in dataset DS1. The alignment depth M is counted after removal of duplicated sequences.

Pfam ID	L	M	PDB ID	Pfam ID	L	M	PDB ID
PF00301	47	4051	1brf	PF00014	53	10628	1aap
PF00048	60	4148	1m8a	PF01363	69	11022	1vfy
PF00542	67	7670	1ctf	PF07647	66	7062	1kw4
PF05198	70	6442	1tif	PF00036	29	5487	1avs
PF00051	79	4526	1i71	PF02214	94	7731	1dsx
PF10150	270	7777	5f6c	PF00030	82	8121	1nps
PF00381	82	9321	1pch	PF00234	87	1871	1fk5
PF04296	75	3552	1g2r	PF01985	84	5759	1jo0
PF00127	99	3946	1ag6	PF00077	101	1313	2hs1
PF02033	105	6787	1jos	PF01807	98	8001	1doq
PF02302	90	9193	1iib	PF07653	57	6654	1i1j
PF01250	90	7316	1vmb	PF00237	103	8664	1i4j
PF00031	92	3341	1roa	PF01668	143	6633	1wjx
PF03960	64	8699	1rw1	PF01152	121	5111	1dlw
PF00238	122	7535	1whi	PF00116	120	7691	2cua
PF02036	101	6687	1c44	PF02579	94	3530	1p9o
PF00572	119	8938	1j3a	PF00241	125	7343	1m4j
PF00565	108	9233	1ihz	PF01389	183	594	1qjp
PF00080	141	6259	1ej8	PF01687	121	7932	1nb9
PF00042	110	7336	1a6m	PF06445	155	6600	1jyh
PF00061	144	4886	1beb	PF00413	158	6350	1hfc
PF02367	128	6576	1htw	PF00959	109	2509	1lpy
PF01812	187	8531	1wkc	PF02233	460	4345	1d4o
PF01195	183	8170	1ryb	PF00186	160	7083	1aoe
PF01661	118	8797	1vhu	PF01339	170	7750	1chd
PF01421	200	6099	1atl	PF04509	38	3446	1xkra
PF00445	185	4081	1dix	PF00045	45	8482	1hxn
PF02224	211	6974	1cke	PF01183	178	5228	1jfx
PF02527	184	6748	1xdz	PF00310	420	7072	1xff
PF00139	249	5676	1gzc	PF01223	226	5147	1ql0

Table B. Dataset DS2: Protein families and PDB structures extracted from the PSICOV benchmark, with MSA sizes obtained from current databases. The alignment depth M is counted after removal of duplicated sequences. The selection criterion was to choose those proteins whose MSAs after removing the duplicate sequences did not exceed 12000 sequences, this led to the 60 proteins presented.

Pfam ID	L	M	PDB ID	Pfam ID	L	M	PDB ID
PF02829	97	967	1j5y	PF01896	182	1770	4bpu
PF12397	116	950	5wy4	PF14720	80	1593	3myr
PF06003	264	1394	4gli	PF03592	141	1072	3zqn
PF04006	617	1570	6nd4	PF02888	75	489	6cno
PF16677	120	185	3p9a	PF17842	147	142	3htx
PF18441	136	131	3htx	PF11053	157	112	3txs
PF08468	157	611	2pjd	PF02723	75	54	5x29
PF02380	93	71	2pf4	PF02315	88	81	5xm3
PF15127	92	109	hvz	PF02287	133	241	1dio
PF16785	111	145	3u8v	PF08640	83	985	5ic8

Table C. Dataset DS3: Protein families and PDB structures with small MSA from current databases. The alignment depth M is counted after removal of duplicated sequences. The selection criterion was to choose Pfam families whose alignments depth varies between 50-1800 sequences and whose structures are well known, this led to the 20 families presented.

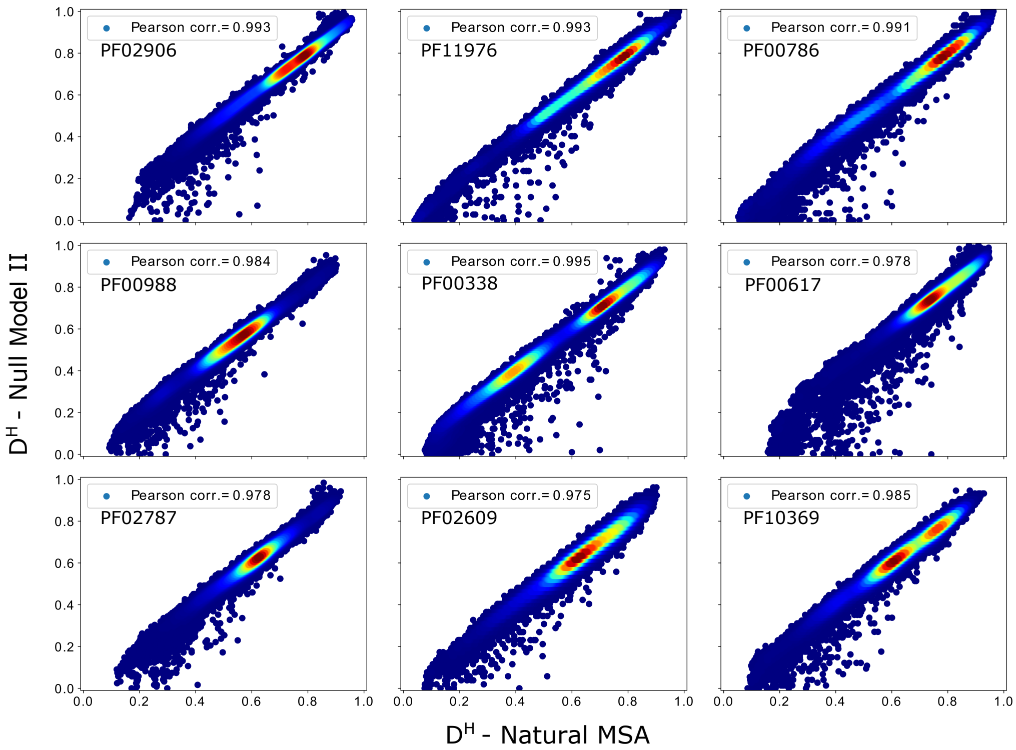

Fig A. Scatter plots of pairwise distances between sequences in the natural MSA vs. an MSA generated by Null model II, for all families in dataset DS1. The inserts show the Pearson correlation between the two distance matrices.

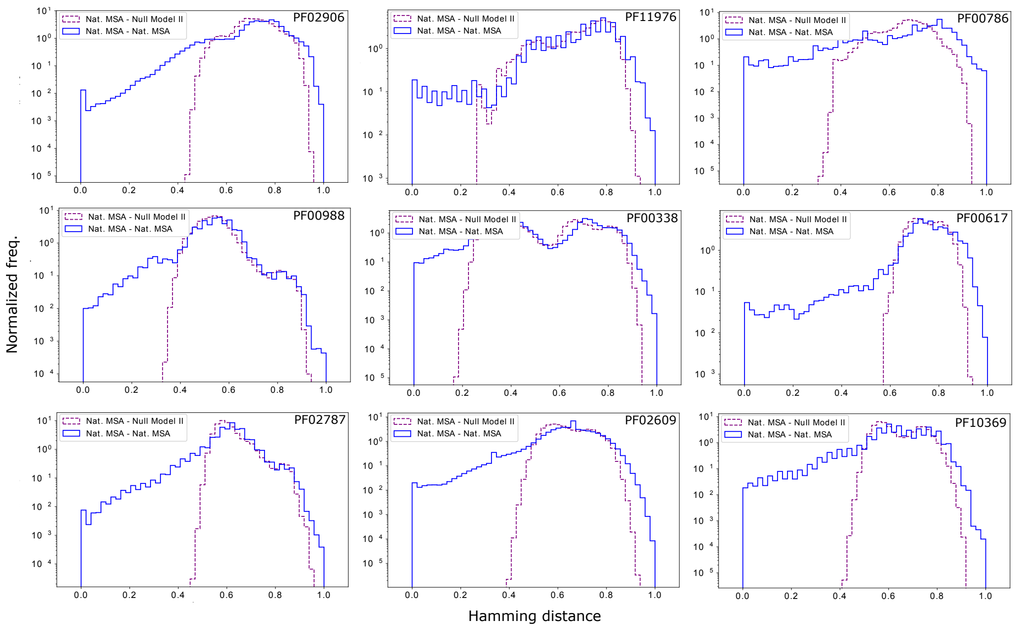

Fig B. Histograms of pairwise distances between sequences in the natural MSA vs. an MSA generated by Null models I and II, for all families in dataset DS1.

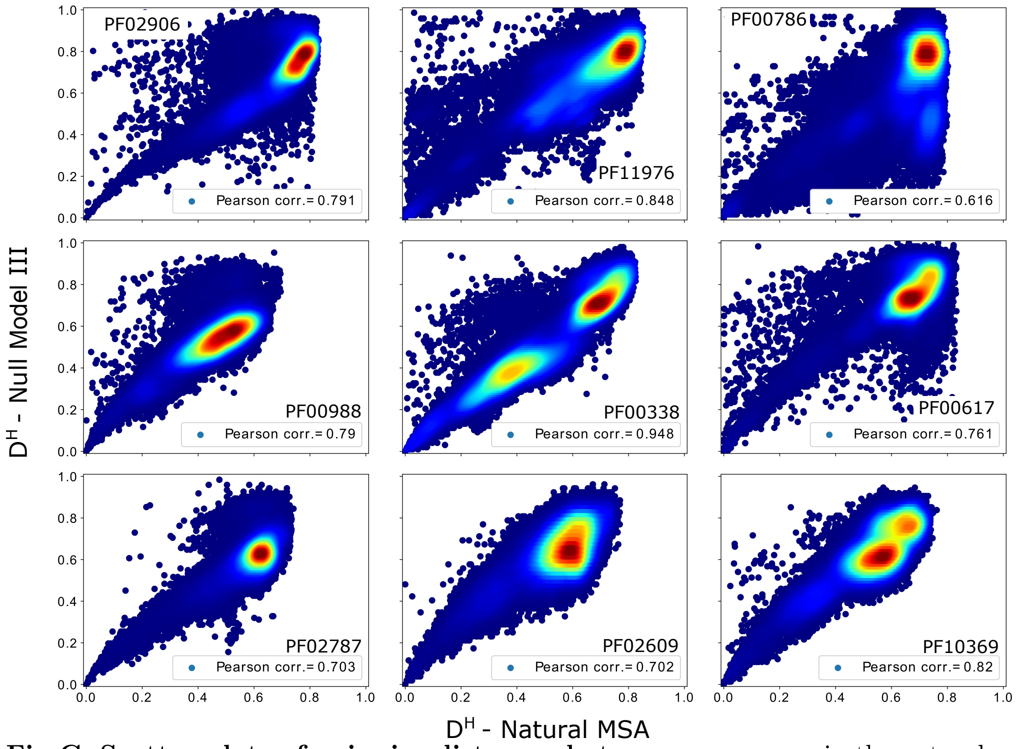

Fig C. Scatter plots of pairwise distances between sequences in the natural MSA vs. an MSA generated by Null model III, for all families in dataset DS1. The inserts show the Pearson correlation between the two distance matrices.

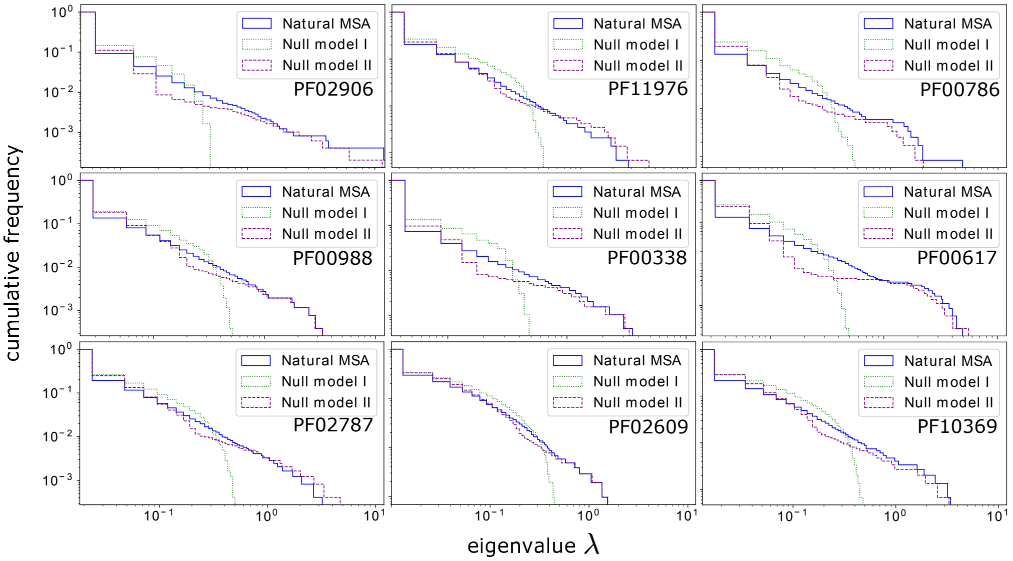

Fig D. Eigenvalue spectra of the covariance matrix of the natural MSA and for Null models I and II: We show cumulative distributions of the eigenvalue spectra for the nine protein families in DS1, i.e. the fraction of eigenvalues larger than λ is shown as a function of λ. We observe that the phylogeny-aware Null model II shows the same fat tail for large eigenvalues, which is also present in the natural data, while the non-phylogenetic Null model I has a more compact support. Data for the Null models are averaged over 50 independent realizations each.

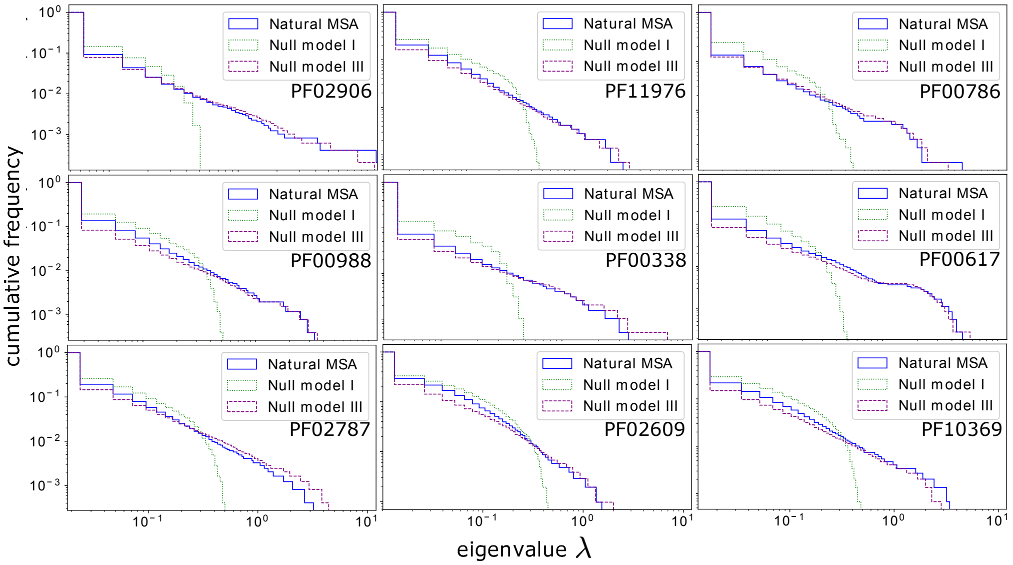

Fig E. Eigenvalue spectra of the covariance matrix of the natural MSA and for Null models I and III: We show cumulative distributions of the eigenvalue spectra for the 9 protein families in DS1, i.e. the fraction of eigenvalues larger than λ is shown as a function of λ. We observe that the phylogeny-aware Null model III shows the same (or an even larger) fat tail for large eigenvalues, which is also present in the natural data, while the non-phylogenetic Null model I has a more compact support. Data for the Null models are averaged over 50 independent realizations each.

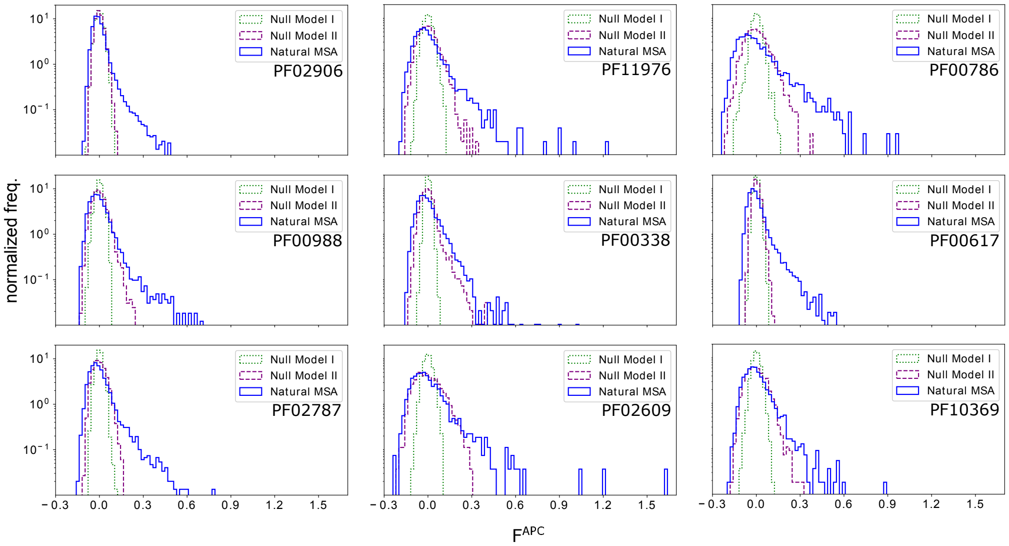

Fig F. Histogram of DCA scores derived from natural sequence data and from MSA generated by Null models I and II : For the 9 protein families in DS1, we show the histograms of DCA coupling scores (APC corrected Frobenius norm of couplings, the standard output of plmDCA), for the natural MSA and samples of Null models I and III. It becomes evident that phylogenetic effects create, to a degree varying from family to family, larger couplings than to be expected from finite sample size alone. However, couplings derived from the natural MSA have substantially larger values.

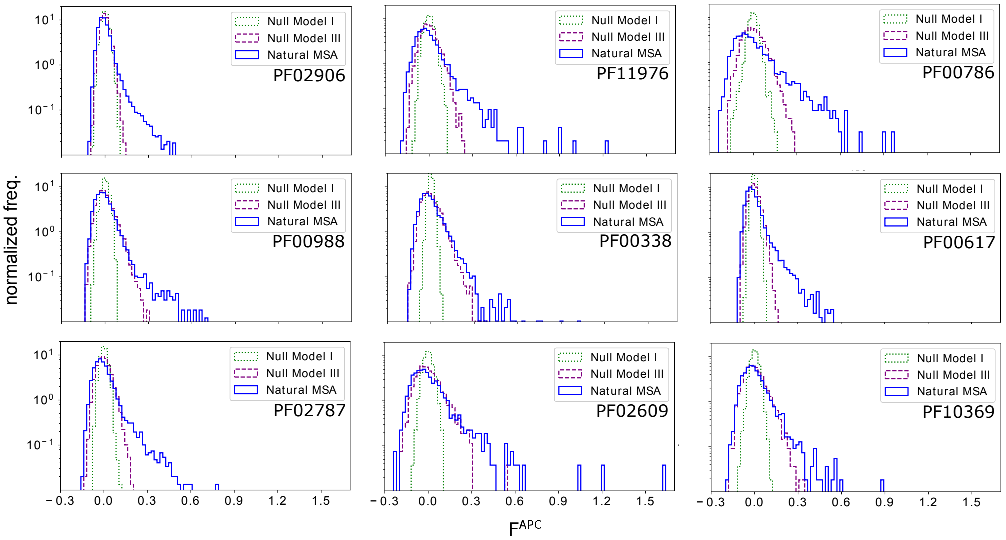

Fig G. Histogram of DCA scores derived from natural sequence data and from MSA generated by Null models I and III : For the protein families in dataset DS1, we show the histograms of DCA coupling scores (APC corrected Frobenius norm of couplings, the standard output of plmDCA), for the natural MSA and samples of Null models I and III. It becomes evident that phylogenetic effects create, to a degree varying from family to family, larger couplings than to be expected from finite sample size alone. However, couplings derived from the natural MSA have substantially larger values.

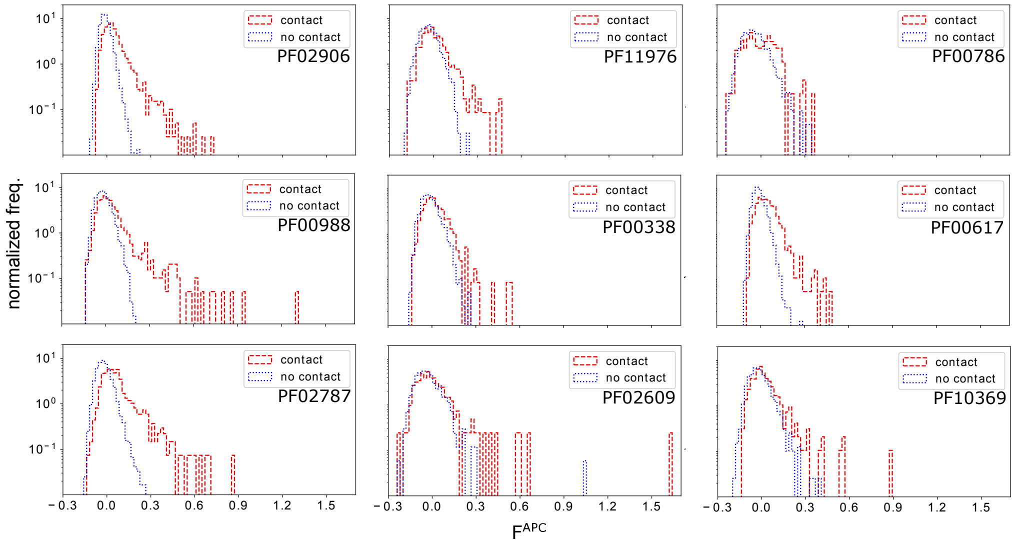

Fig H. Histogram of DCA scores derived from natural sequence data for residue-residue contacts and non-contacts: For the protein families in dataset DS1, we show the histograms of DCA coupling scores (APC corrected Frobenius norm of couplings, the standard output of plmDCA), separated for contacts and non-contacts. Only pairs with linear separation $|i - j| > 4$ along the chain are taken into account. It is clear that for scores above about 0.2-0.3, most predictions are true contacts (false positives may actually be oligomeric contacts), and the quality of contact prediction mostly depends on how many predictions above this threshold are found in a protein family.

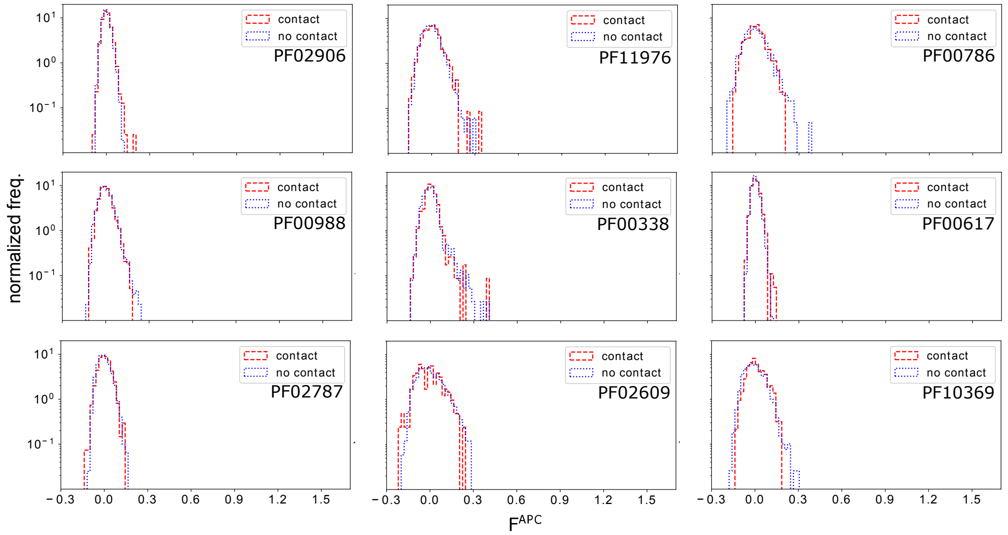

Fig I. Histogram of DCA scores derived from Null model II for residue-residue contacts and non-contacts: For the protein families in dataset DS1, we show the histograms of DCA coupling scores (APC corrected Frobenius norm of couplings, the standard output of plmDCA), separated for contacts and non-contacts. Only pairs with linear separation $|i - j| > 4$ along the chain are taken into account. It becomes evident that any signal related to contacts is totally destroyed by the randomization procedure in Null model II. Interestingly, the null model generates almost no couplings with scores above 0.2-0.3, which was seen in Fig. H as a cutoff for high-accuracy contact prediction.

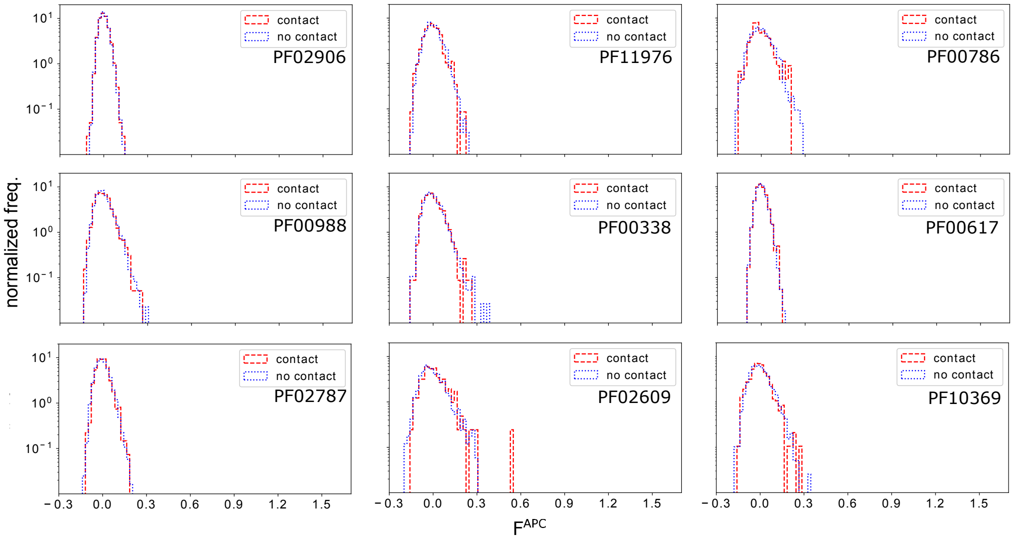

Fig J. Histogram of DCA scores derived from Null model III for residue-residue contacts and non-contacts: For the protein families in dataset DS1, we show the histograms of DCA coupling scores (APC corrected Frobenius norm of couplings, the standard output of plmDCA), separated for contacts and non-contacts. Only pairs with linear separation $|i - j| > 4$ along the chain are taken into account. It becomes evident that any signal related to contacts is totally destroyed by the randomization procedure in Null model III. Interestingly, the null model generates almost no couplings with scores above 0.2-0.3, which was seen in Fig. H as a cutoff for high-accuracy contact prediction.

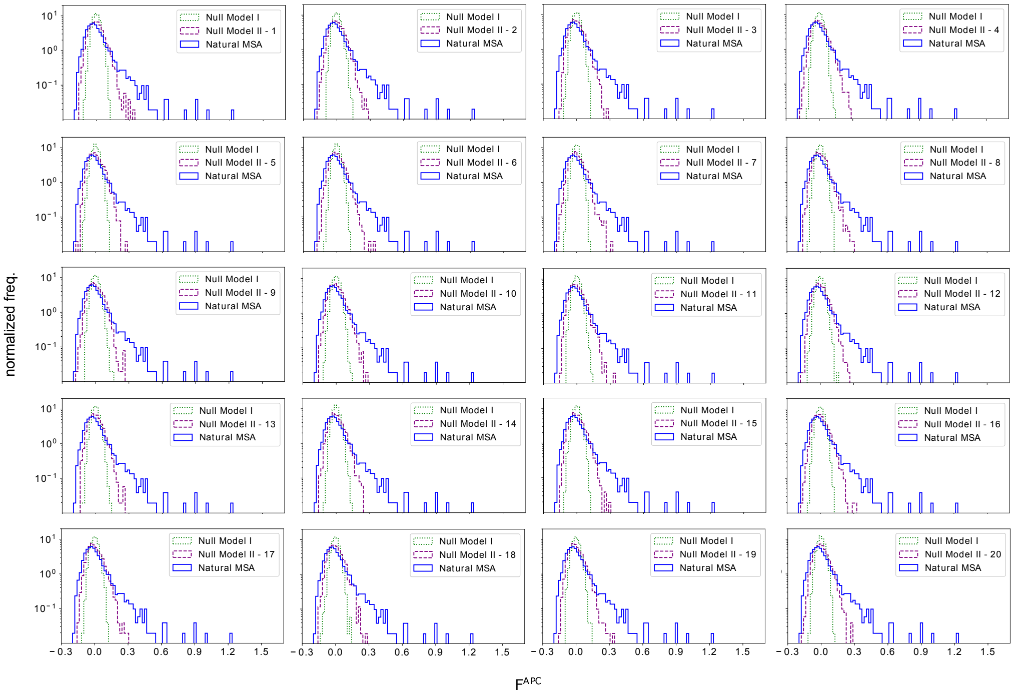

Fig K. Histograms of DCA scores derived from 20 independent samples of Null model II for protein family PF02906: The histograms of couplings are robust with respect to sample-to-sample fluctuations of Null model II.

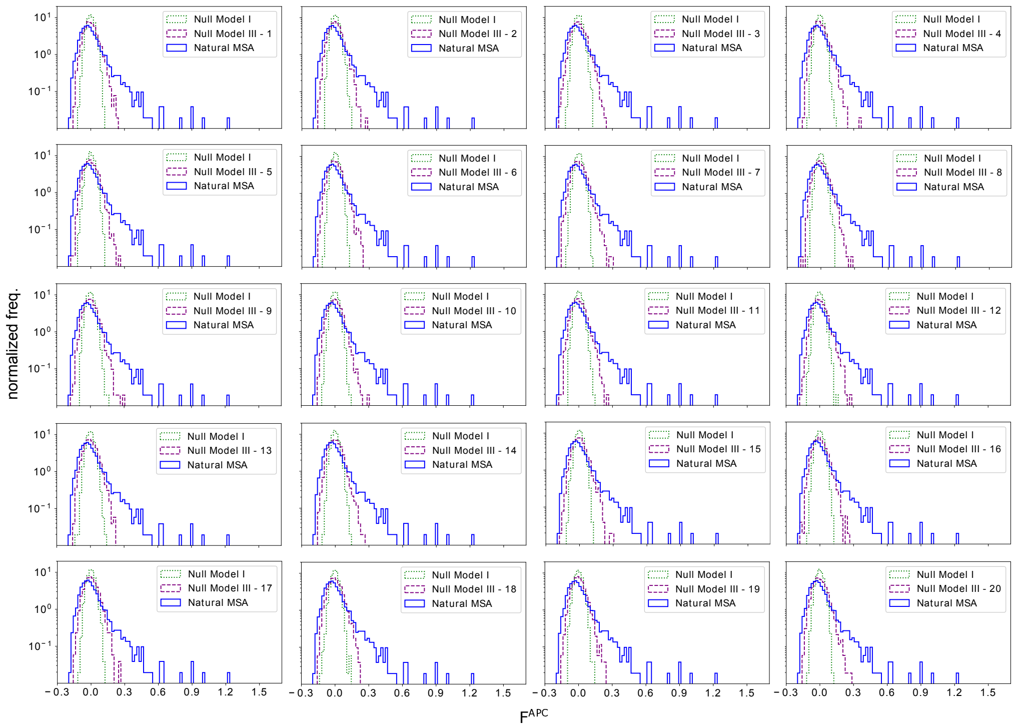

Fig L. Histograms of DCA scores derived from 20 independent samples of Null model III for protein family PF02906: The histograms of couplings are robust with respect to sample-to-sample fluctuations of Null model III.

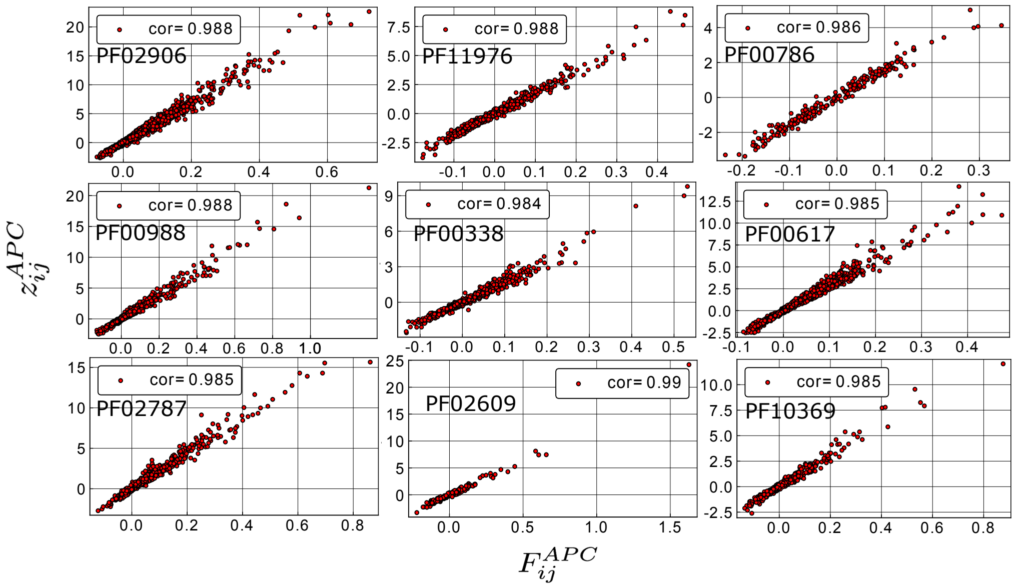

Fig M. z-scores of couplings derived from the natural MSA, as compared to the distribution of couplings derived from Null model III: For each residue pair (i, j), we calculate the z-score for the DCA score derived from natural data as compared to 50 realizations of Null model III. Results are shown for dataset DS1.
