# Supplementary material for: On the effect of phylogenetic correlations in coevolution-based contact prediction in proteins
Source: PLoS Comput Biol. 2021 May 24;17(5):e1008957. doi: 10.1371/journal.pcbi.1008957 (PMC8177639; doi:10.1371/journal.pcbi.1008957)
Supplement: Attachment — Submitted filename: reviewer_reply.pdf [file pcbi.1008957.s002.pdf]

We thank all reviewers for the careful reading of our manuscript, which has helped us to substantially improve our results and presentation. We apologize for the long time it took is to revise the manuscript, but the authors were (and partially are) confined on different continents – the first author in a country with very limited private access to the internet and thus to the computing resources in the other author's lab – has substantially slowed down our work. We hope that our revised version answers all questions and criticisms of the reviewers, and allows for a publication of our manuscript in PLoS Computational Biology.

The most important changes are an extension of the number of protein families from 9 to now 89 families, containing in particular a dataset with 20 families having small-to-medium size multiple sequence alignments. The extension has allowed us to have more robust support for our conclusions, and also the better study the relative influence of finite dataset size vs. phylogenetic correlations.

We also would like to mention, that our method has been used by one of us (ERH) in a collaboration with E. Aurell and other colleagues, which was searching for epistatic couplings between mutations in more than 50,000 SARS-Cov-2 genomes. Thanks to the application of Null model II, the authors were able to establish the existence of a sparse but statistically signification network of epistatic couplings, and Null model II was essential for the assessment of statistical significance. The paper is published: [Zeng, Hong-Li, Vito Dichio, Edwin Rodríguez Horta, Kaisa Thorell, and Erik Aurell. "Global analysis of more than 50,000 SARS-CoV-2 genomes reveals epistasis between eight viral genes." PNAS 117 (2020): 31519-31526].

Our replies to the reviewers are given below. The original reports are included in black, our answers in blue.

We hope therefore that the method itself can now be accepted for publication in PLoS Comp Biol.

Reviewer #1: Coevolution analysis forms the basis of many recent methods for template-free protein structure prediction. It aims to predict 3D contacts between residues in protein domains from their statistical signature of coevolution. All methods in use today treat the sequences in the input multiple sequence alignment (MSA) as being independent, with some simple sequence weighting to correct for different degrees of redundancy. This is a gross assumption because in fact the protein sequences are statistically dependent on each other, as described by the phylogenetic tree that can be reconstructed from the MSA.

This study investigates the influence of the assumption of independence by randomizing real MSAs such that the sequence composition at each position is conserved and the pairwise distances between the sequences is conserved, while the statistical signature of co-evolution signatures between residues is remove (except for noise that remains, of course).

The main results of the study are reported to be
(1) that spurious co-evolution signatures caused by the unjustified approximation of independence are not limiting our ability to predict contacts for the most strongly co-evolving pairs of positions;
(2) that we could predict many more contacts if we were able to suppress the spurious couplings by a proper treatment of the phylogenetic dependence of the sequences.

Main issues:

1. Result (1) is not as general as the authors purport. It only applies to MSAs with very many sequences and as such is close to self-fulfilling prophecy. If the MSAs are large enough, the phylogenetic noise will not limit the contact predictions. Also, these results are not new and not surprising. We have known for a long time that the position pairs obtaining the top DCA scores in large MSAs are to a large fraction in contact, except for false positives caused by homo-multimerization and other well-described effects. Other studies have shown that the independence assumption adds considerable noise to the coupling scores.

We fully agree that it is well known that almost all large couplings correspond to contacts, as is shown in particular in Refs. [38.39]. However, there is some threshold below which most couplings start to be false positives in terms of contact prediction, even if homo-oligomerization or couplings mediated by ligands or other interaction partners are taken into account. The reasons for this failure might be various: not all contacts coevolve, not all contacts are important (and thus constrained) in all sequences, finite MSA lead to non-zero noisy couplings etc. What we show is that couplings generated by phylogeny are **quantitatively** coherent with this threshold. We have made this more clear in the revised manuscript, both in the discussions, but also in combining coupling histograms with positive-predictive value curves in Fig. 3.

2. To learn a huge number of coupling parameters without overfitting, the models such as GREMLIN and DCA use strong regularizing square penalties than push the coupling parameters towards zero. MSAs with many diverse sequences therefore produce higher scores, as the signal can better push against the strong regularizers. Therefore, the larger and more diverse the MSA is, the larger the top co-evolution scores

usually are and the less limiting the influence of spurious signals from phylogeny will be. Therefore the depth and diversity of the MSA is an absolutely critical parameter to understand the influence and limitations induced by the independence assumption. The authors have ignored this parameter.

Also, they show results only for very large MSAs (table 1), large enough to predict with current methods a sufficient number of contacts reliably to predict their structures reliably. The interesting regime is of course that of an intermediate number of sequences, 50- 500 for example, for which it is still challenging to predict contacts.

We thank the reviewer for bringing up this point; we agree that this was missing in our original submission. We have now included also an analysis of smaller MSA (dataset DS3). In these cases we see that the phylogenetic noise is only marginally stronger than the finite-sample noise in the couplings, i.e. also a clever method removing phylogenetic biases cannot be expected to improve contact prediction. However, we also see that the phylogeny-induced couplings are relatively robust with respect to the sample size, while the finite-sample effects decrease when larger MSA are considered, and the coevolutionary contact-signal increases.

3. So what? It is not clear in what way the insights in this study would help us to improve contact prediction in the future. How precisely might we reduce or remove the spurious signals from the unjustified assumption of independence of the sequences? How can we use the presented null models to improve contact prediction?

The objective of the paper was not to solve the problem of phylogenetic biases in DCA-type models, but to quantitatively assess if the problem actually exists and a solution might help to extract more contacts from sequence data. This is, in our perception, an interesting basic-science question, and the results seem clear to us. Solving this problem, i.e. designing a coevolutionary method taking phylogenetic effects out of the direct couplings, remains a hard task. Please remember that basically all phylogenetic methods use an independent-site approximation, all coevolutionary methods an independent-sequence approximation (up to some empirical corrections), but both types of signal coexist in the data and should be considered together. We think that our paper shows that it might be worth the effort, in particular in the case of sufficiently large MSA.

To add, our Null Model II has found a first application in [Zeng, Hong-Li, Vito Dichio, Edwin Rodríguez Horta, Kaisa Thorell, and Erik Aurell. "Global analysis of more than 50,000 SARS-CoV-2 genomes reveals epistasis between eight viral genes." PNAS 117 (2020): 31519-31526], where it is used in collaboration with one of us (ERH) to show that observable epistatic couplings between mutations in SARS-Cov-2 are not compatible with phylogenetic effects; this point was essential for that paper to be accepted in PNAS. We hope that this illustrates that the method is actually useful in real applications (which in the case of the mentioned paper go beyond contact prediction).

Reviewer #2: This is an interesting and insightful paper.
It tackles a long-standing issue in DCA, namely the relevance of phylogenetic correlations between sequences and how they affect the inferred co-evolutionary coupling strengths.

The authors address the problem elegantly, and their main result is that, mostly, phylogenetic correlations affect only low-strength couplings, so that high-strength ones can be safely trusted.

Although their work is convincing, I have some issues that I would like to see addressed.

We thank the reviewer for the positive assessment of our work, and we hope that our revision answers fully to his questions.

1) As a practitioner, I (and I guess most people using DCA for practical applications) know that DCA works extremely well, with really very very few false positives within the first N contacts (N being the length of the sequences in the MSA). Once down-weighted by similarity, thus, it seems that phylogenetic correlations play only a minor role. This is perfectly in line with their findings.

My understanding is that correlated mutations for O(N) pairs give the same final scores whether they are "clustered" on branches of the tree or scattered everywhere. And in principle, the only reason why we should downplay their role when the same pair appears in a subbranch is not because they are close on the tree, but because the full sequences are close to each other (otherwise they would be wonderful co-occurrences to be duly appreciated by DCA). But this sequence similarity is precisely what re-weighting accounts for.

In this respect, I have to admit that I never felt the worry about phylogenetic correlations as compelling and urgent (interesting for sure to understand, but not necessarily as a major concern).

Could the authors comment?

We agree with the reviewer that in many practical applications reweighting leads to satisfying results. However, reweighing is a very simple heuristic scheme accounting for a single distance scale between sequences, which is set by hand via the reweighting threshold. Phylogenetic trees have branchings at all distances, and have a complex structure, which cannot be captured by simple reweighting.

We think therefore that it is an interesting academic question to assess the influence of phylogenetic correlations in DCA-type methods. Besides this basic-science aspect, it has also practical applications. One of us (ERH) has applied Null model II to assess epistatic couplings found between mutations in SARS-Cov-2, and his findings where essential for the work to get accepted for publication: [Zeng, Hong-Li, Vito Dichio, Edwin Rodríguez Horta, Kaisa Thorell, and Erik Aurell. "Global analysis of more than 50,000 SARS-CoV-2 genomes reveals epistasis between eight viral genes." PNAS 117 (2020): 31519-31526].

2) Connected to this, I'd also like to comment on the null models they used in this manuscript, to understand to what extent theirs is not a circular argument.

My understanding is that phylogenetic trees are built using the independent site hypothesis. Some of them are built using (weighted) Hamming distances, others using likelihood approaches. In this respect, what the authors do is perfectly consistent with the correlation present in these trees. Nonetheless, that's the definition of phylogeny that people has given because i) it works and ii) it is mathematically and computationally tractable. Should the phylogenetic information that really affects DCA be encoded in ways that escape the independent site approximation used to build trees, it would not be addressed by the present manuscript.

In this respect they might more carefully phrase their results stating that phylogeny information related to tree-building is only mildly affecting the results, whereas deeper phylogenetic information, beyond the single site approximation, might still do it and this should be addressed in future work.

This is just a suggestion to stress that what is real (phylogenetic correlation) is different from the approximate ways we use to capture it (present tree-building algorithms).

This is an interesting question, which concerns in particular Null model III (in a certain sense also Null model II since based on Hamming distances). We actually have the impression that the fact that phylogenies are reconstructed using the assumption of independent-site evolution even strengthens our Null model III: at no point, not even in the reconstruction of the phylogenetic tree, coevolution between residues has been taken into account. So our Null model III corresponds fully to a situation where **all** correlations in the re-emitted MSA come from either finite size or the tree. We have stated this more clearly in our revised manuscript.

Basically we ask: assuming independent-site evolution at all levels, what are the spurious couplings found by DCA as compared to the couplings found on real data.

Reviewer #3: In this paper authors performed an analysis based on Null models to support the idea that phylogenetic correlations only provide a limited contribution to coevolution-based contact prediction methods.

To prove their claim, authors defined two types of Null models preserving conservation only and conservation+phylogeny from the original MSA and compared DCA coupling obtained with these models against those derived from the original MSA. As result, they show that, while phylogenetic effects are dominant to determine the structure of the residue-residue correlation matrix, coupling derived by DCA using MSAs preserving conservation+phylogeny but not containing any coevolution signal are not sufficient to distinguish residue-residue contacts.

Overall, I believe that the procedure applied is sound and results are properly supported by data. However, I suggest to better justify the choice of 9 protein families analyzed in this study, possibly providing more details on data selection. In general, I believe that enlarging the set of considered families would definitely add to the paper. Maybe, results for some additional family could be reported and discussed in the supplementary material.

We thank the reviewer for the overall positive assessment of our work. We agree that 9 proteins are a bit limited datasets, and therefore we have extended the dataset using part of the PSICOV benchmark data, and including also small MSA, which where not present in our original selection. Due to the limited access of ERH to computational resources during lockdown (Cuba has limited access to private internet, and the computational resources are located in France), but also due to the fact that our Null models become very expensive in case of very deep MSA (Null model II reconstructs M*(M-1)/2 distances between M sequences), we have limited the overall dataset now to about 89 protein families with a maximum of 12,000 sequences per family. The main results remain robust, but they get refined in the case of smaller MSA, which were not included in the original 9-family dataset.

Minor:
I suggest to reproduce all figures to improve readability in B/W
There is a missing citation in Fig4 caption

We have revised the figures, in particular the histograms, and corrected the caption. The figures are now well readable in B/W, but the use of different line stiles makes them a bit less clear in colors. We would be happy to adapt further modifications in the figure styles if needed.

Reviewer #4: The authors investigate the influece of phylogenetic correlations in coevolutionary-based contact prediction. The effect of phylogentic correlations is investigated by analyzing the performances of plmDCA (a coevolutionary-based contact predictor) on real MSAs against those obtained on the same MSAs after a reshuffling that keeps unaltered the position specific amino acid frequecies and the pairwise Hamming distances between sequences (i.e. Null model II).

As a general comment, the approach looks interesting although I find it hard to understand whether the conclusions are interesting or not. My major concers follow.

1. The authors evaluate DCA couplings at sequence separation > 4. Such sequence separation is probably too short to get strong conclusions from the analysis. Residue-residue contacts are more abundant for short sequence separations and are usually ignored since they "mask" the more interesting long-range contact. In fact, contact prediction accuracy is usually assessed on long range contacts (sequence separation > 24), which provide stronger constraints on the protein 3D structure.

In the coevolutionary literature, sequence separation above 4 is widely used, so we would like to stick to this choice in the main text. We have, however, repeated our analysis for sequence separation above 24, and we find very similar results, as is shown in the new Fig. 3 of our manuscript. The main problem with sequence separation is that there are less contacts and less predictions, so the statistics is worse. However, the positive-predictive values in function of the DCA-score F^APC are almost unchanged, with a slightly larger region of transition from low to high PPV.

2. It is not clear why the author chose to focus only on 9 Pfam protein families. Since this work essentually provides a statistical analysis, more robust conclusions may be obtained on a larger set of protein families. For example, I would suggest to consider the (quite popular) benchmark set of 150 single chain, single domain proteins taken from the PSICOV/MetaPSICOV benchmark dataset (obtained from Pfam). Also, the author should find some way to summarize all the results in a single plot/table instead of showing a different plot for each protein family. As an alternative, the authors should at least justify why this cannot be done or why this is not useful/interesting.

We agree that 9 proteins are a bit limited datasets, and therefore we have extended the dataset using part of the PSICOV benchmark data, and including also small MSA, which where not present in our original selection. Due to the limited access of ERH to computational resources during lockdown (Cuba has limited access to private internet, and the computational resources are located in France), but also due to the fact that our Null models become very expensive in case of very deep MSA (Null model II reconstructs M*(M-1)/2 distances between M sequences), we have limited the overall dataset now to about 89 protein families with a maximum of 12,000 sequences per family, including also 20 smaller MSA. The main results remain robust, but they get refined in the case of smaller MSA, which were not included in the original 9-family dataset.

We have also moved the presentation from showing different plots for individual families to showing summary results, only distinguishing between larger and small MSA depths. We have moved many of the individual plots to the Supplement, since they show the variability of the phylogeny-induced signal from family to family; this information would be lost in the summary figures.

3. In order to make the results more accessible to the scientific community that works on protein contact prediction it would be useful to to compare the contact prediction accuracy on the real MSAs against the accuracy obtained on the shuffled MSAs (e.g. Null model II). In particular, it is not clear whether plmDCA achieves good or bad prediction accuracy on the benchmark set.

We have included a new Fig. 5 with positive predictive values (PPV) for standard plmDCA, the Null models II, and the z-scores (cf. the reviewers question 5). We see that the null models bring contact prediction almost down to a random prediction; the little deviation is due to the fact that conservation is also included in our Null models, and more conserved residues appear in highly non-random positions in the proteins (e.g. active sites or the protein core). These figures, and the inclusion of PPV in Fig. 3 in function of the DCA score, should render the results more accessible for the community interested in contact prediction.

4. The conclusions are a bit misleading. For instance, in Section “Conclusion" the authors state that "contact prediction is influenced only to a very limited degree by phylogenetic couplings" but they also add that "it is

also striking that, across several protein families, the phylogeny-caused couplings in Null models II and III almost reach the DCA-score threshold found before for accurate contact prediction. This suggests that the suppression of phylogenetic biases in the data (or their better consideration in model inference), may shift this threshold down and therefore allow for predicting much more contacts.” First of all, I remark again that 9 protein families are not enough to draw strong conlusions. Anyway, such concluding remarks do not clarify whether we should care or not about phylogenetic bias in coevolutionary-based contact prediction.

We have reformulated large parts of the conclusions and of the discussion of our results, after having reinforced them by including more protein families and adding a dataset with smaller MSA. We hope that this reformulation meets the criticism of the reviewer.

5. It would be interesting to know (or at least discuss) whether the phylogeny-caused couplings identified on the shuffled MSAs can be used to filter-out false positive contact predictions. Maybe I am worng, but at least in principle, it seems to me that this could be possible by simply performing a Z-test.

We have used z-scores for contact prediction, the results seem highly compatible with standard DCA scores using APC-corrected Frobenius norms of the coupling matrices between residue positions. This is not totally surprising seen the correlation between coupling scores and z-scores. Due to the fact that z-scores do not improve contact prediction, we had not included the curves in our original submission, but we agree that the results are, even if not positive, an important result of our work, cf. Fig. 5.

In the meantime, one of us (ERH) has applied Null model II to assess epistatic couplings found between mutations in SARS-Cov-2, and his findings were essential for the work to get accepted for publication: [Zeng, Hong-Li, Vito Dichio, Edwin Rodríguez Horta, Kaisa Thorell, and Erik Aurell. "Global analysis of more than 50,000 SARS-CoV-2 genomes reveals epistasis between eight viral genes." PNAS 117 (2020): 31519-31526].

6. I would be curious to know how much the null model II shuffles the single protein sequences. Such analysis coulbe easily done by simply comparing/aligning a sequence in the shuffled MSA against those in the real MSA in order to detect the one with the highest sequence similarity. The average sequence similarity can thus give an idea of how much the shuffled MSA is similar to the real one.

We thank the reviewer for bringing this up. We have included the supplementary Fig, S2 measuring pairwise distances between natural and shuffled sequences. We see that shuffled sequences are typically at least 30-40% away from any natural sequence. This is a good test that constraining distances does not lead to close-to-natural sequences – this observation would have partially invalidated our conclusions.

Note that shuffled sequences respect conservation and are therefore recognized as “homologs” by profile-based alignment methods like HMMer.
