# Supplementary material for: On the effect of phylogenetic correlations in coevolution-based contact prediction in proteins
Source: PLoS Comput Biol. 2021 May 24;17(5):e1008957. doi: 10.1371/journal.pcbi.1008957 (PMC8177639; doi:10.1371/journal.pcbi.1008957)
Supplement: Attachment — Submitted filename: reviewer_reply.pdf [file pcbi.1008957.s003.pdf]

The original reviewer's comments are shown in black, our replies in blue.

I thank the authors for the clearer statement of their conclusions and for the improvements of their analyses despite the difficulties of working with bad Internet connections etc. As such the study sheds light on an interesting and not much quantitatively researched aspect of Potts models.

We thank the reviewer for the appreciation of our revised manuscript, the comments of all four reviewers have made our paper more solid and better understandable. We thank the reviewer also for bringing up some more points, which we have carefully considered in our second revision, as is detailed below in our point-by-point answer. We also add a pdf with highlighted changes. We hope that this clarifies the last open issues, and that our paper becomes now acceptable for publication in PLoS Computational Biology.

I have two remaining major issues that should be easy to address.

Major point:

1. Starting on line 393, the authors write:
`Suppression of phylogenetic biases in the data (or their better consideration in model inference) ... may allow for predicting much more contacts. The comparison with Null model I, which contains only finite-sample effects at given residue conservations, shows that sufficiently big protein families will be needed to exploit this. For the smallest protein families, represented by our dataset DS3, the finite-sample effects are found to be almost as large as the phylogenetic effects on the DCA results, while the two were well separated in the case of the deeper 400 MSA in datasets DS1 and DS2.'.

Looking at Fig. 3B (showing data for DS3 with small MSAs), at F^APC value corresponding to a PPV of 0.5, the density of null model I predictions in green is about a factor 2 lower than the density of null model II predictions. This means if the phylogenetic biases could be removed, the PPV would increase from TP/(TP+FP) = 0.5 to roughly TP'/(TP'+FP) = TP/(TP+FP/2) = 0.67. This is no small improvement and contradicts the above statement made by the authors.

Obviously, this result is somewhat surprising as the logarithmic y axis hides the magnitude of the effect, and it is cumbersome to read it off the graph. It would therefore be important to add traces to Fig. 5 that show the PPV versus number of predictions for the hypothetical case that phylogenetic effects can be corrected, for DS2 and DS3. The discussion and conclusions will probably have to be adjusted accordingly.

We thank the reviewer for bringing up this excellent question and suggesting a hypothetical curve without phylogenetic effects in Fig. 5. To do so, we have kept the original DCA scores on all contacts, but we have replaced scores for the non-contacting residue pairs with their values in a realisation of Null model I, i.e., by taking into account only finite-size effects on these pairs. In both DS2 and DS3, the PPV are clearly increased. However, we observe for the large MSA in DS2 a clear plateau at average PPV close to one (Panel C), while in DS3 the average PPV is increased as expected by the reviewer, but it does not reach one. This shows that a successful removal of phylogenetic effects would even help in small MSA.

We have modified the figure and the discussion accordingly.

2. Did the authors use sequence weighting? For the comparison of the green and magenta traces in Fig. 3 it is strictly necessary to not use sequence weighting. Otherwise, the weights for null model I would be close to one (since sequences are independent) while those of null model II would be significantly smaller than 1, which would systematically shrink the sizes of the coupling coefficients under null model II and make the distribution incomparable to null model I. Sequence weighting would also render the analysis in point 1 meaningless.

We did not use reweighting. Like the reviewer, we think that this empirical correction would confound our analysis. We have now clearly stated that no sequence weights are used.

Minor points:

3. Define abbreviation APC, explain in 1-2 sentences what it does and give a proper reference for completeness.

Now we introduced the abbreviation APC in the introduction, where we already had the original reference and a short discussion of the average-product correction. Later, when using APC in our results, we refer to the introduction.

4. Define `normalized frequency' used in Fig. 3.

Histograms are normalised as probability distribution functions, i.e. to having area one under the curve. This makes histograms robust with respect to binning. We have defined this in the revised caption of Fig. 3, i.e., at first occurrence.

5. The caption of Figure 4 is missing an explanation what A and B refers to.

We have corrected the caption.

6. In figure 4B, what sense does contact and non-contact make for null model II where there are no contacts?

Since all null models keep MSA width and single-residue conservation statistics, they are easily mapped to representative structures of the original biological MSA. This mapping defines pairs in contact and non-contact. We have clarified this definition in our revised manuscript.

7. Line 363:
`In Fig. 5 we show that the two lead to highly comparable results, with a slight advantage for the standard F^APC after the first few predictions.'
Please explain why. My guess: Residue conservation is positively correlated with both higher variance of the DCA scores and with a higher probability for a contact. Therefore, dividing by the square root of the variance can reduce PPV.

We do not have a good explanation, but the idea suggested by the reviewer is quite convincing, also because there is some structural signal even in null models, mainly because of the non-random positioning of conserved positions in the protein structure. We have included the idea, marking it as a somewhat speculative interpretation.

We hope that our new revision answers all questions, and that the manuscript can now be accepted for publication.
